# Supplementary material for: Transcript profiling of plastid ferrochelatase two mutants reveals that chloroplast singlet oxygen signals lead to global changes in RNA profiles and are mediated by Plant U-Box 4
Source: BMC Plant Biol. 2025 Jun 3;25:747. doi: 10.1186/s12870-025-06703-7 (PMC12131553; doi:10.1186/s12870-025-06703-7)
Supplement: Supplementary file 1 — Additional file 1: Supplemental information, Figures S1 through S15. Figure S1. Tetrapyrrole profiling of fc2 mutants, Figure S2. Visualization of variance among RNA-seq replicates used in the study, Figure S3. Volcano plot analysis of mean differential gene expression between genotypes in a single condition, Figure S4. Gene ontology analyses of differentially expressed genes in fc2 mutants, Figure S5. Analysis of stress marker gene expression in the single pub4 mutant, Figure S6. Gene ontology analyses of differentially expressed genes in fc2 pub4, Figure S7. WRKY, ERF, and NAC transcription factor networks are induced by chloroplast singlet oxygen accumulation, Figure S8. Gene Ontology term enrichment of differentially expressed transcription factors genes and transcription factor gene families. Figure S9, WRKY and NAC transcription factor targets are induced by chloroplast singlet oxygen accumulation and activation of NAC targets is blocked by the pub4 mutation. Figure S10, Gene ontology analyses of differentially expressed plastid protein-encoding genes in fc2 mutants, Figure S11. Gene ontology analyses of differentially expressed plastid protein-encoding genes shared between fc2 and fc2 pub4 mutants, Figure S12. Gene ontology analyses of differentially expressed plastid protein-encoding genes unique to fc2 pub4 mutants, Figure S13. Relative expression of plastid protein-encoding genes during singlet oxygen stress, Figure S14. Analysis of salicylic acid response marker gene expression in the single pub4 mutant, Figure S15. Activation of senescence pathways is blocked by the pub4 mutation. [file 12870_2025_6703_MOESM1_ESM.pdf]

Supplemental information, Figures S1 through S15.

This file contains the following Supplemental Figures, each on a separate page:

Figure S1. Tetrapyrrole profiling of *fc2* mutants.

Figure S2. Visualization of variance among RNA-seq replicates used in the study.

Figure S3. Volcano plot analysis of mean differential gene expression between genotypes in a single condition.

Figure S4. Gene ontology analyses of differentially expressed genes in *fc2* mutants.

Figure S5. Analysis of stress marker gene expression in the single *pub4* mutant.

Figure S6. Gene ontology analyses of differentially expressed genes in *fc2 pub4*.

Figure S7. WRKY, ERF, and NAC transcription factor networks are induced by chloroplast singlet oxygen accumulation.

Figure S8. Gene Ontology term enrichment of differentially expressed transcription factors genes and transcription factor gene families.

Figure S9. WRKY and NAC transcription factor targets are induced by chloroplast singlet oxygen accumulation and activation of NAC targets is blocked by the *pub4* mutation.

Figure S10. Gene ontology analyses of differentially expressed plastid protein-encoding genes in *fc2* mutants.

Figure S11. Gene ontology analyses of differentially expressed plastid protein-encoding genes shared between *fc2* and *fc2 pub4* mutants.

Figure S12. Gene ontology analyses of differentially expressed plastid protein-encoding genes unique to *fc2 pub4* mutants.

Figure S13. Relative expression of plastid protein-encoding genes during singlet oxygen stress.

Figure S14. Analysis of salicylic acid response marker gene expression in the single *pub4* mutant.

Figure S15. Activation of senescence pathways is blocked by the *pub4* mutation.

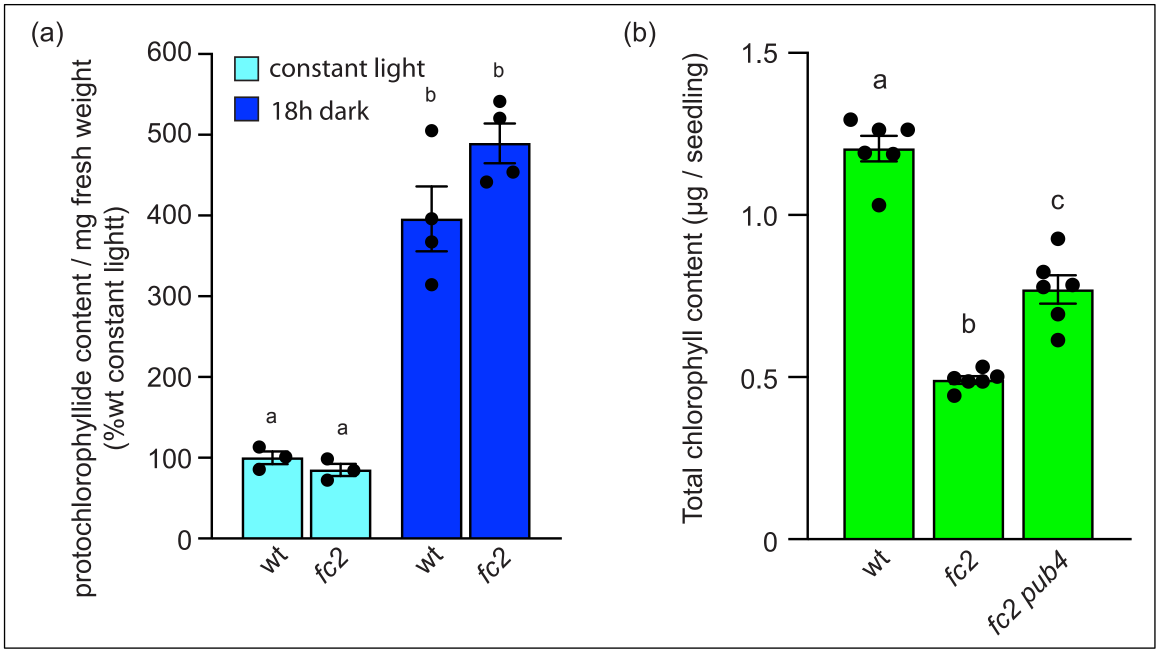

<u>Figure S1. Tetrapyrrole profiling of *fc2* mutants</u>.
Protochlorophyllide and total chlorophyll content was determined in wt and *fc2* plants. (a) Mean protochlorophyllide levels (%wt in constant (24h) light) (+/- SEM) of seven-day-old seedlings grown under constant (24h) light conditions for six days and then incubated in the dark for 18 hours (18h dark). As a control, another set of seedlings was kept in constant light conditions (constant light) ($n \geq 3$ biological replicates). (b) Mean total chlorophyll content (µg / seedling) (+/- SEM) of five-day-old seedlings grown in 24h light conditions ($n = 6$ biological replicates). Statistical analyses were performed by a one-way ANOVA test followed by Tukey's HSD. Letters indicate statistically significant differences between samples ($P \leq 0.05$). Closed circles represent individual data points.

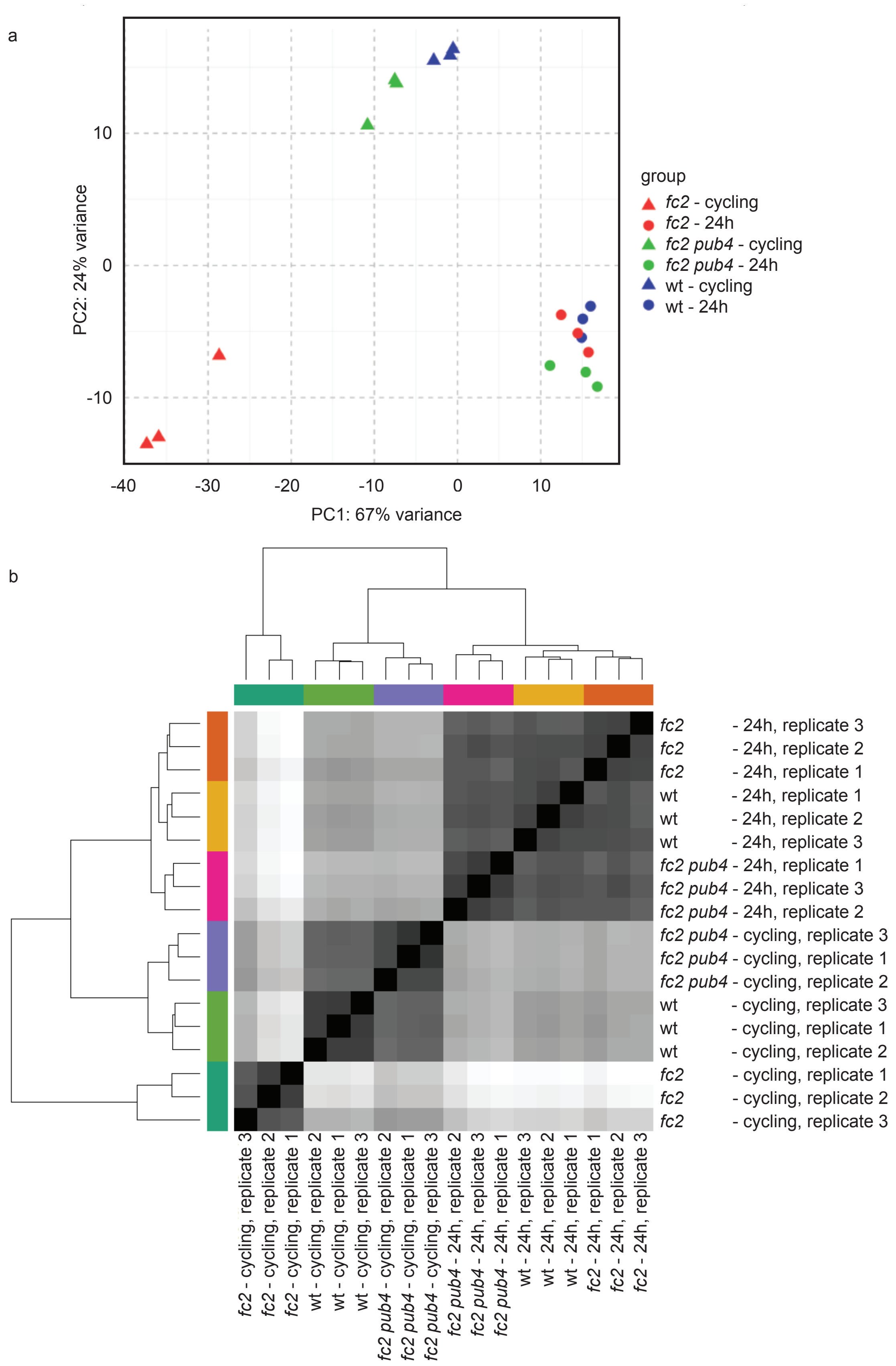

<u>Figure S2. Visualization of variance among RNA-seq replicates used in the study.</u>

Shown are two analyses to visualize variance among the 18 RNA-seq replicates used in this study. Three genotypes (wt, *fc2*, and *fc2 pub4*) were grown in two conditions (19 days in constant light conditions (24h) or 17 days of constant light conditions followed by two days on 16h light/8h dark diurnal cycling light conditions (cycling)) for a total of 6 groups with three replicates each. (a) Principal component analysis (PCA) scatter plot showing variance of the RNA-seq replicates. Principal component (PC) 1 and PC2 are plotted and colored by genotype (wt, *fc2* and *fc2 pub4*) and growing condition (constant light (24h) and 16h light/8h dark diurnal cycling light (cycling) conditions) for each replicate (3 each). The genotypes and conditions are color coded according to the key on the right. PCA was performed using feature count matrix produced from the RNA-seq data sets. Variation percentage for each PC (PC1: 67% variance, PC2: 24% variance) reported in brackets with the axis label. (b) Shown is a distance matrix for the same 18 replicates. The heatmap was generated using feature count matrix produced from the RNA-seq data sets and the clustering of samples show an overview of similarities and dissimilarities between the replicates. Shades of grey indicate distance between samples with darker shades indicating smaller distance and lighter shades indicating greater distance.

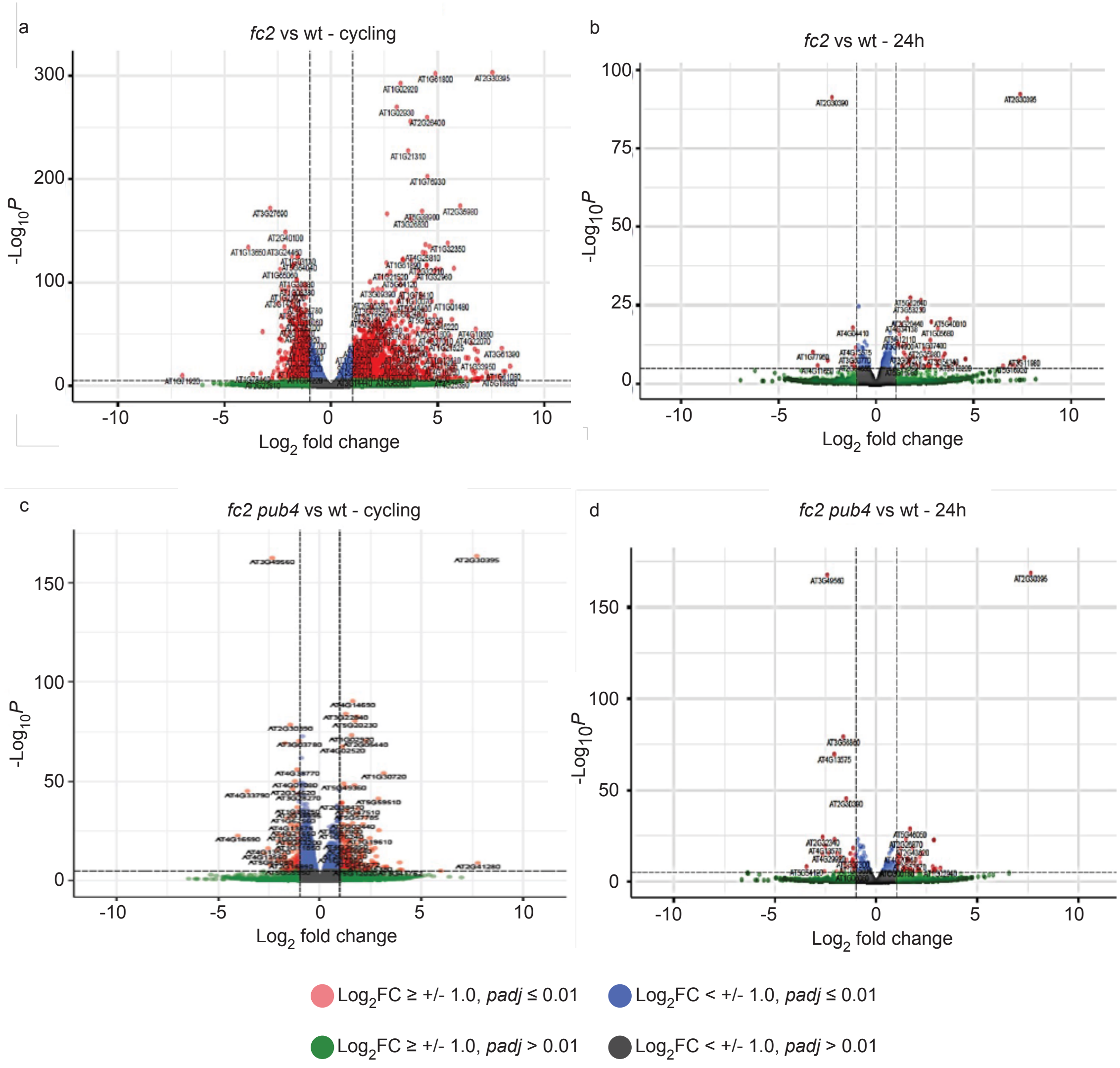

<u>Figure S3. Volcano plot analysis of mean differential gene expression between genotypes in a single condition.</u>

Volcano plots showing pairwise comparisons between genotypes (wt vs. *fc2* or wt vs. *fc2 pub4*) within a single condition (permissive constant light (24h) conditions or two days of singlet oxygen-producing 16h light/8h dark diurnal cycling light conditions (cycling)). Expression data is from the DESeq2 analysis of the included RNA-seq data set; (a) *fc2* vs. wt in cycling light conditions, (b) *fc2* vs. wt in 24h light conditions, (c) *fc2 pub4* vs. wt in cycling light conditions, and (d) *fc2 pub4* vs. wt in 24h light conditions. The y-axis represents the negative log_{10}-transformed *P*-values from gene-specific tests, and the x-axis shows the log_2 fold change (FC). Red dots represent differentially expressed genes (DEGs) according to the log_2FC and *padj* cut-off values of ≥ +/-1 and ≤ 0.01 respectively. Green dots represent DEGs that only pass the log_2FC cutoff, blue dots represent DEGs that only pass the log_2FC cutoff, and dark grey dots represent DEGs that did not pass either cutoff. The most upregulated genes are towards the right, the most downregulated genes are towards the left, and the most statistically significant genes are towards the top.

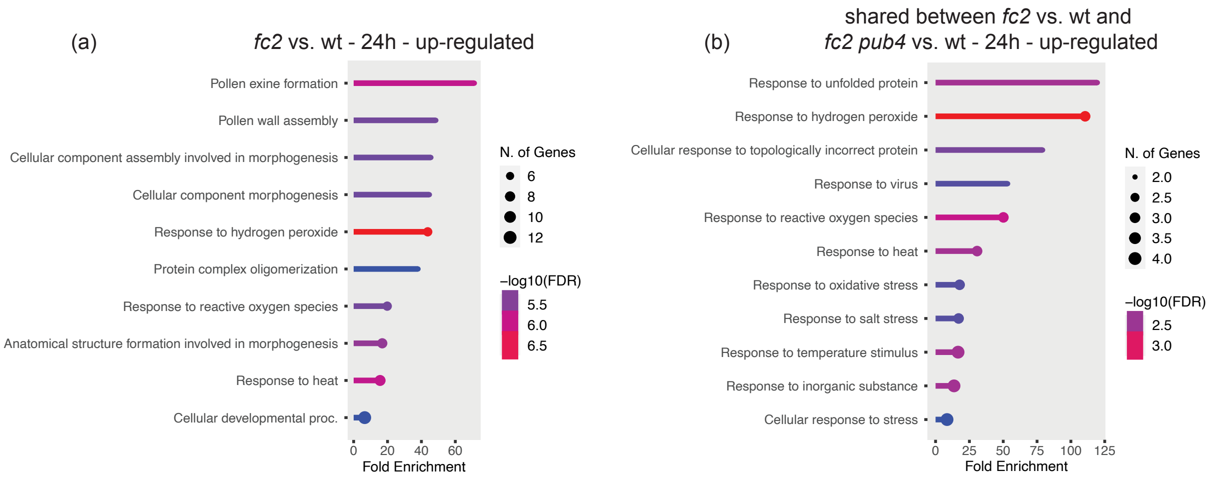

Figure S4. Gene ontology analyses of differentially expressed genes in *fc2* mutants.
Shown are gene ontology (GO) term enrichment analyses of the identified differentially expressed genes (DEGs) in this study. (a) GO-term enrichment from the 69 up-regulated DEGs from *fc2* vs. wt under constant (24h) light conditions. (b) GO-term enrichment from the 11 shared up-regulated DEGs between *fc2* vs. wt and *fc2 pub4* under constant (24h) light conditions. GO-term enrichment analyses were performed using ShinyGO 0.82 with an FDR cutoff of $p \leq 0.01$. x-axes indicate fold-enrichment. The ball size indicates number of genes. The line colors represent FDR values.

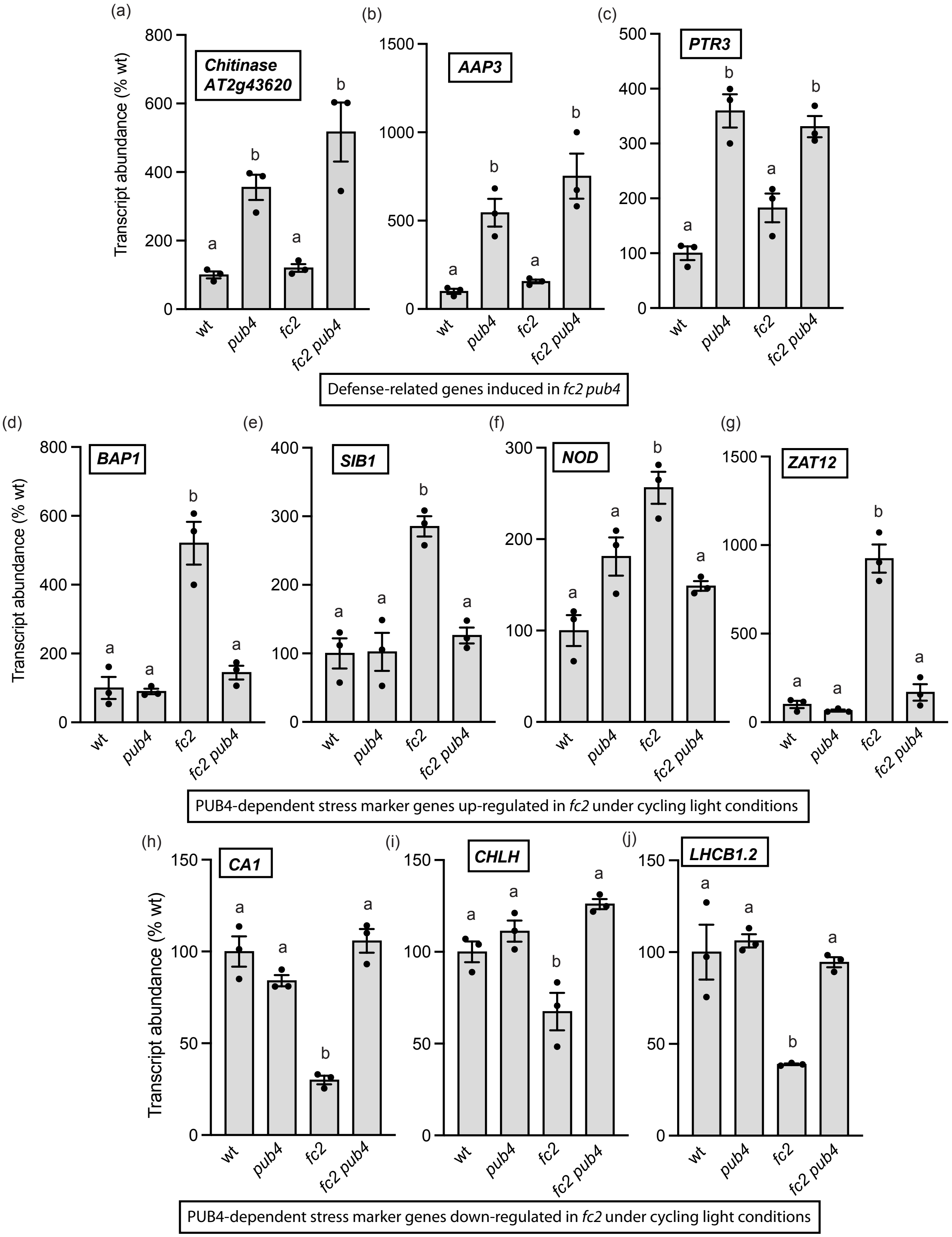

Figure S5. Analysis of stress marker gene expression in the single *pub4* mutant.

Shown are transcript profiles from 21-day-old plants as measured by RT-qPCR. (a-c) Transcript levels of defense-related genes (*Chitinase AT2g43620*, *AAP3*, *PTR3*) in plants grown under 24h

constant light conditions. (d-g) Transcript profiles of PUB4-dependent stress marker genes (*BAP1*, *SIB1*, *NOD*, *ZAT12*) that are up-regulated in *fc2*. Plants were grown for 19 days in 24h constant light conditions and shifted to cycling light conditions (16h light / 8h dark) for two days and harvested one hour after subjective dawn. (h-j) Transcript profiles of PUB4-dependent stress marker genes (*CA1*, *CHLH*, *LHCB1.2*) that are down-regulated in *fc2*. The same cDNA probed in panels d through g was used. Shown are mean values compared to *ACTIN2* expression (+/- SEM) and normalized to wt (n = 3 plants [total above ground tissue]). Statistical analyses were performed using one-way ANOVA tests, and the different letters above the bars indicate significant differences within data sets determined by Tukey-Kramer post-tests ($P \leq 0.05$). Closed circles indicate individual data points.

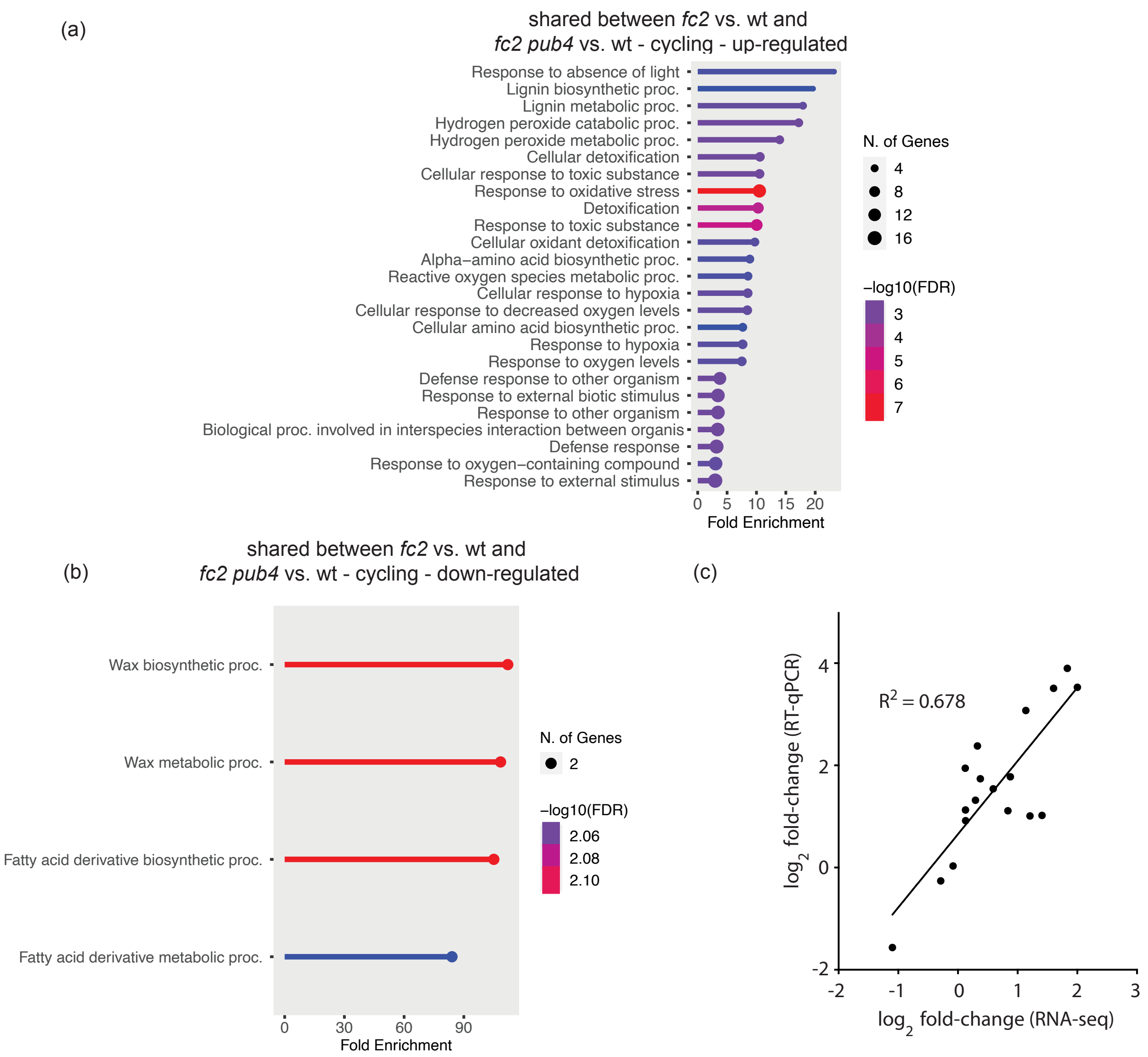

<u>Figure S6. Gene ontology analyses of differentially expressed genes in *fc2 pub4*.</u>

Shown are gene ontology (GO) term enrichment analyses of the identified differentially expressed genes (DEGs) in this study. (a) GO-term enrichment from the 80 shared up-regulated DEGs between *fc2* vs. wt and *fc2 pub4* under 16h light/8h dark diurnal cycling light (cycling) conditions. (b) GO-term enrichment from the 80 shared down-regulated DEGs between *fc2* vs. wt and *fc2 pub4* under 16h light/8h dark diurnal cycling light (cycling) conditions. GO-term enrichment analyses were performed using ShinyGO 0.80 with an FDR cutoff of $p \leq 0.01$. x-axes indicate fold-enrichment. The ball size indicates number of genes. The line colors represent FDR values. (c) RNA-seq analysis of plants grown in cycling light conditions was confirmed by RT-qPCR using RNA extracted from an independent set of plants grown in the same conditions (17 days of 24h constant light conditions, followed by two days of 16h light/8h dark diurnal cycling light conditions and harvested one hour post dawn). Shown is the correlation of independent RNA-seq and RT-qPCR analyses of pairwise comparisons between wt and *fc2* under cycling light conditions and wt and *fc2 pub4* under cycling light conditions. The fold change values (log_2) of nine nuclear

genes (*SIB1*, *PAO*, *CHLH*, *LOX4*, *AOC3*, *EDS16*, *CAM1*, *GPA1*, *PAP1*) are plotted (total of 18 comparisons). Expression values normalized to expression of *ACTIN2*.

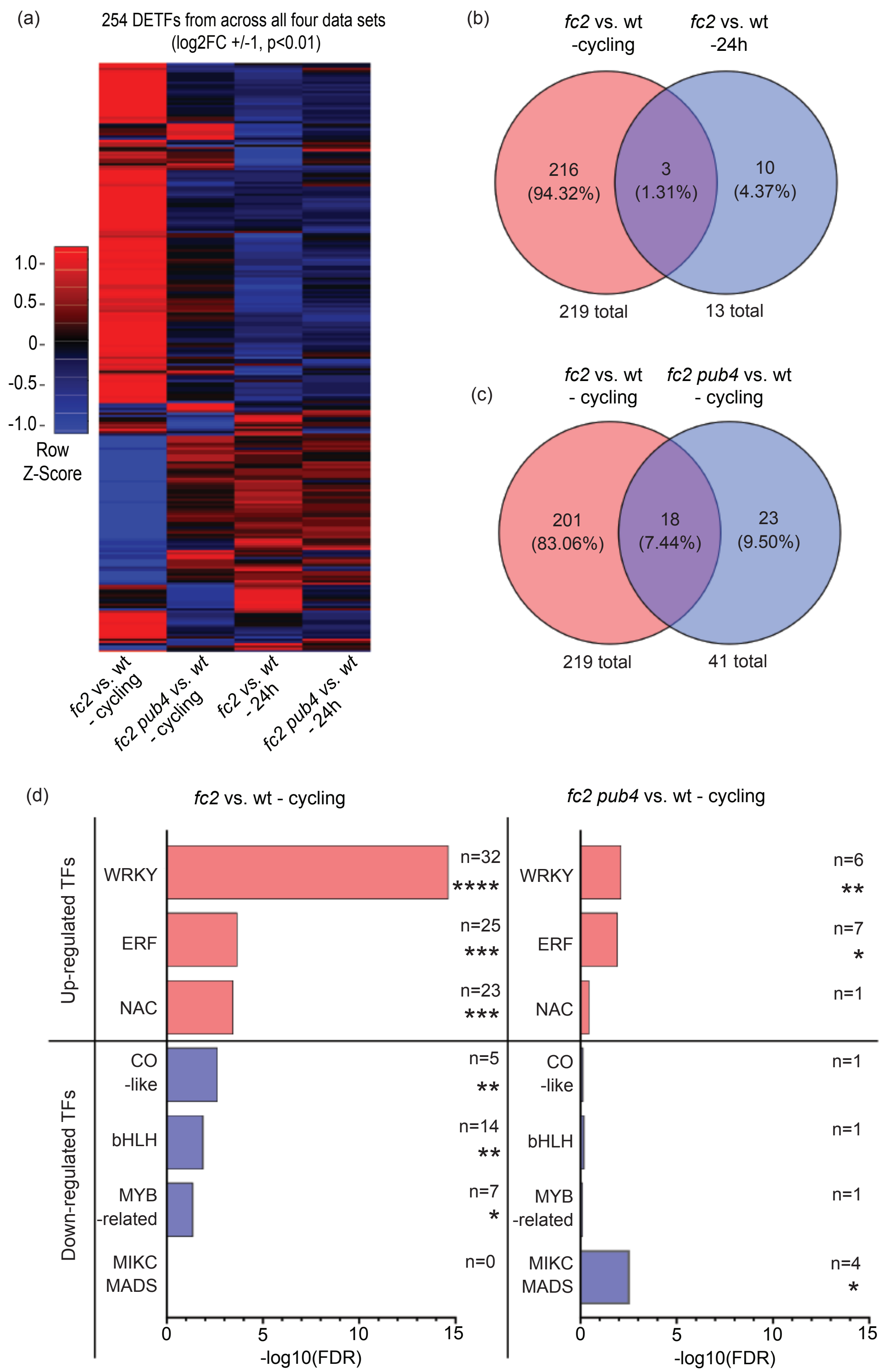

Figure S7. WRKY, ERF, and NAC transcription factor networks are induced by chloroplast singlet oxygen accumulation.

The RNA-seq data set was analyzed for differentially expressed transcription factors (DETFs). (a) A heatmap showing the change in expression of 254 DETFs identified from all differentially expressed genes (DEGs) lists from pairwise comparisons between wt and the mutants (*fc2* or *fc2 pub4*) in each condition (constant light conditions (24h) or 16h light/8h dark diurnal cycling light conditions (cycling) (cutoffs ≥ +/- 1.0 log_2FC, *padj* ≤ 0.01). Red color indicates up-regulation, blue indicates down-regulation, and black indicates no change. (b) Venn diagram comparing the DETFs identified in DEG lists obtained from "*fc2* vs. wt – cycling" (219 genes) and "*fc2* vs. wt - 24h" (13 genes). (c) Venn diagram comparing the DETFs identified in DEG lists obtained from "*fc2* vs. wt – cycling" (219 genes) and "*fc2 pub4* vs wt – cycling" (41 genes). (d) Enrichment of up-regulated (red bars) and down-regulated (blue bars) DETF families from "*fc2* vs. wt – cycling" and "*fc2 pub4* vs. wt – cycling" analyses. n represents the count of respective TF family genes represented in each list. A hypergeometric test followed by the Benjamini–Hochberg method was used to determine the False Discovery Rate (FDR) (* = $P \leq 0.05$, ** = $P \leq 0.01$, *** = $P \leq 0.001$, **** = $P \leq 0.0001$).

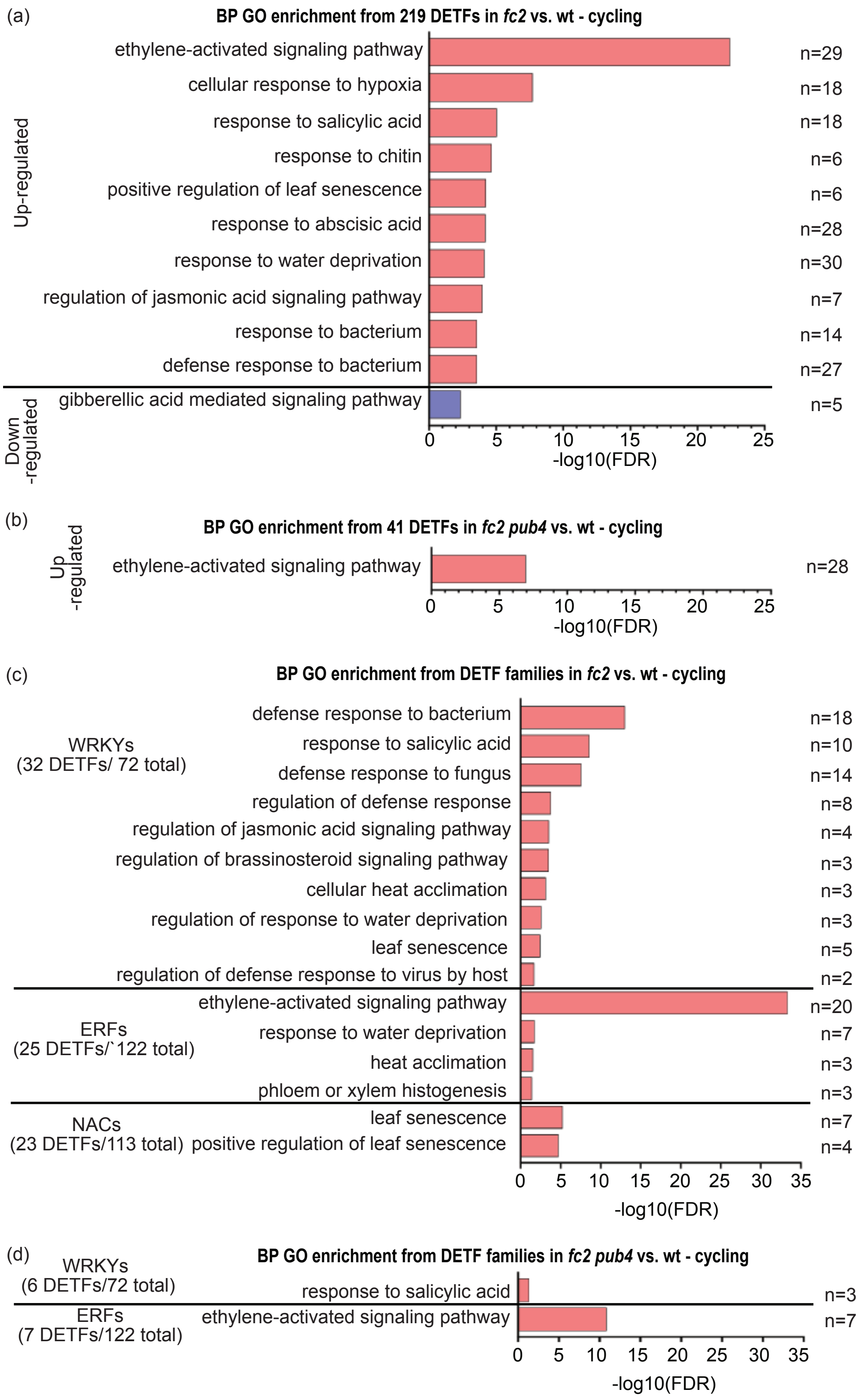

<u>Figure S8. Gene Ontology term enrichment of differentially expressed transcription factors genes and transcription factor gene families.</u>

Differentially expressed transcription factor (DETF) and DETF families from the "*fc2* vs. wt – cycling" (219 genes) and "*fc2 pub4* vs. wt – cycling" (41 genes) pairwise comparisons were assessed for biological process (BP) gene ontology (GO) term enrichment. (a) BP GO term enrichment from the "*fc2* vs. wt – cycling" pairwise comparison (219 genes). (b) BP GO terms enrichment from the "*pub4* vs. wt -cycling" pairwise comparison (41 genes). (c) BP GO term enrichment of WRKY (32 genes), ERF (25 genes), and NAC (23 genes) transcription factor (TF) family genes up-regulated in the "*fc2* vs. wt – cycling" pairwise comparison. (d) BP GO term enrichment of WRKY (32 genes) and ERF (25 genes) TF family genes up-regulated in the "*fc2 pub4* vs. wt – cycling" pairwise comparison. Red bars = up-regulated and blue bars = down-regulated, respectively. GO term enrichment analysis was performed using the Database for Annotation, Visualization, and Integrated Discovery (DAVID) 2021 (https://david.ncifcrf.gov/). Top 10 significant terms (where applicable) are reported. n represents the count of respective TF or TF family genes represented in each list. An FDR ≤ 0.05 cutoff was applied to enriched GO terms found in this analysis.

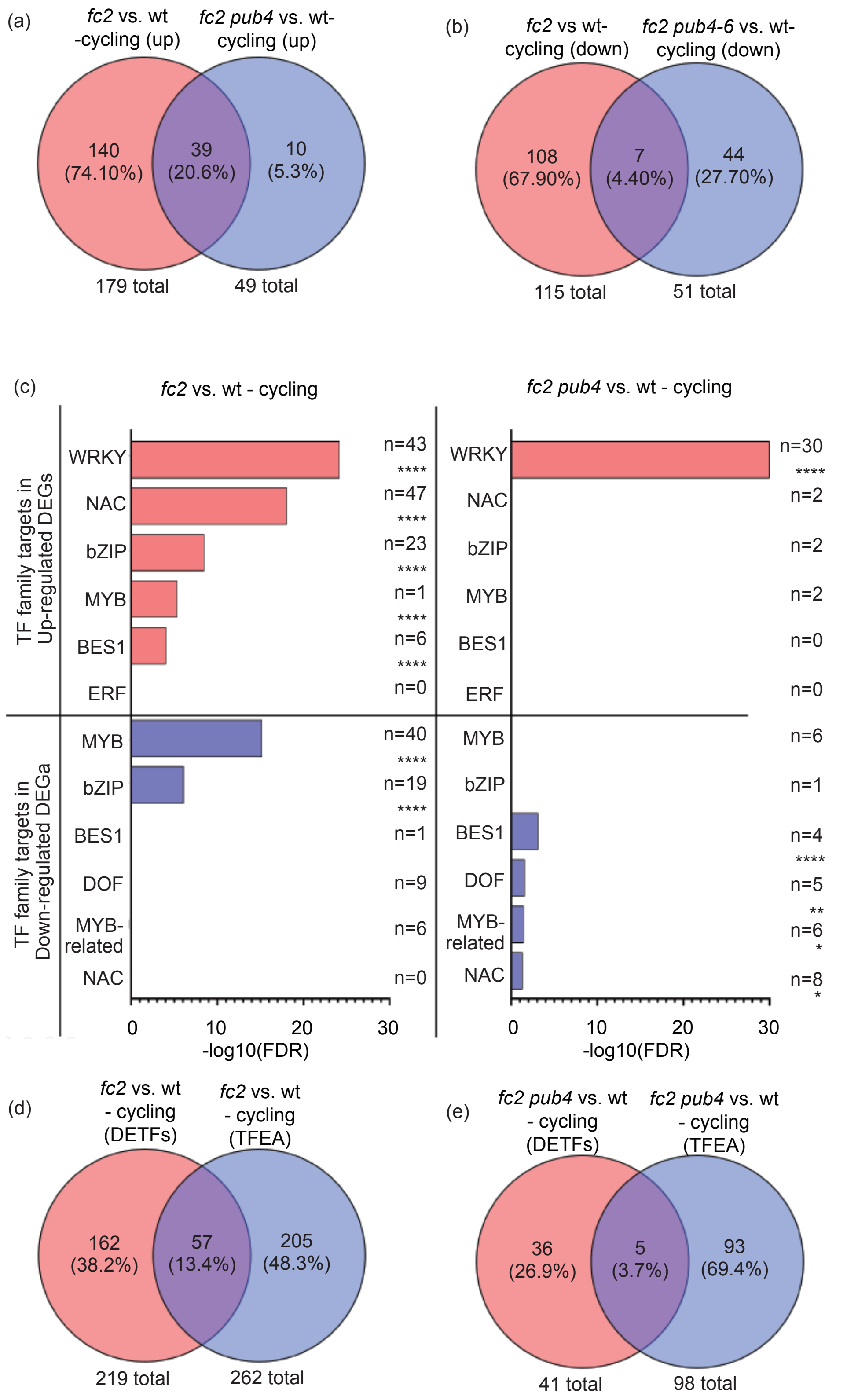

Figure S9. WRKY and NAC transcription factor targets are induced by chloroplast singlet oxygen accumulation and activation of NAC targets is blocked by the *pub4* mutation.

Transcript levels determined by RNA-seq analyses was used to determine the transcription factors (TFs) whose targets were enriched in chloroplast singlet oxygen ($^1O_2$) signaling. Venn diagrams depicting the transcription factor enrichment analyses (TFEAs) which identified the TFs with enriched targets in (a) up-regulated and (b) down-regulated genes identified in differentially expressed gene (DEG) lists obtained from "*fc2* vs. wt – cycling" and "*fc2 pub4* vs. wt – cycling" pairwise comparisons. (c) Enrichment of TF families from "*fc2* vs. wt – cycling" and "*fc2 pub4* vs. wt – cycling" pairwise comparisons whose targets were significantly enriched in their corresponding DEG lists. Red bars = up-regulated and blue bars = down-regulated, respectively. n represents the count of respective TF family genes represented in each list. A hypergeometric test followed by the Benjamini–Hochberg method was used to determine the False Discovery Rate (FDR) (* = $P \leq 0.05$, ** = $P \leq 0.01$, *** = $P \leq 0.001$, **** = $P \leq 0.0001$). Venn diagram comparing the DETFs identified in **Figs. S7b and c** with TFs obtained from the TFEA using up- and down-regulated DEGs from (d) "*fc2* vs. wt – cycling" (262 TFs) and (e) "*fc2 pub4* vs. wt – cycling" (98 TFs) pairwise comparisons.

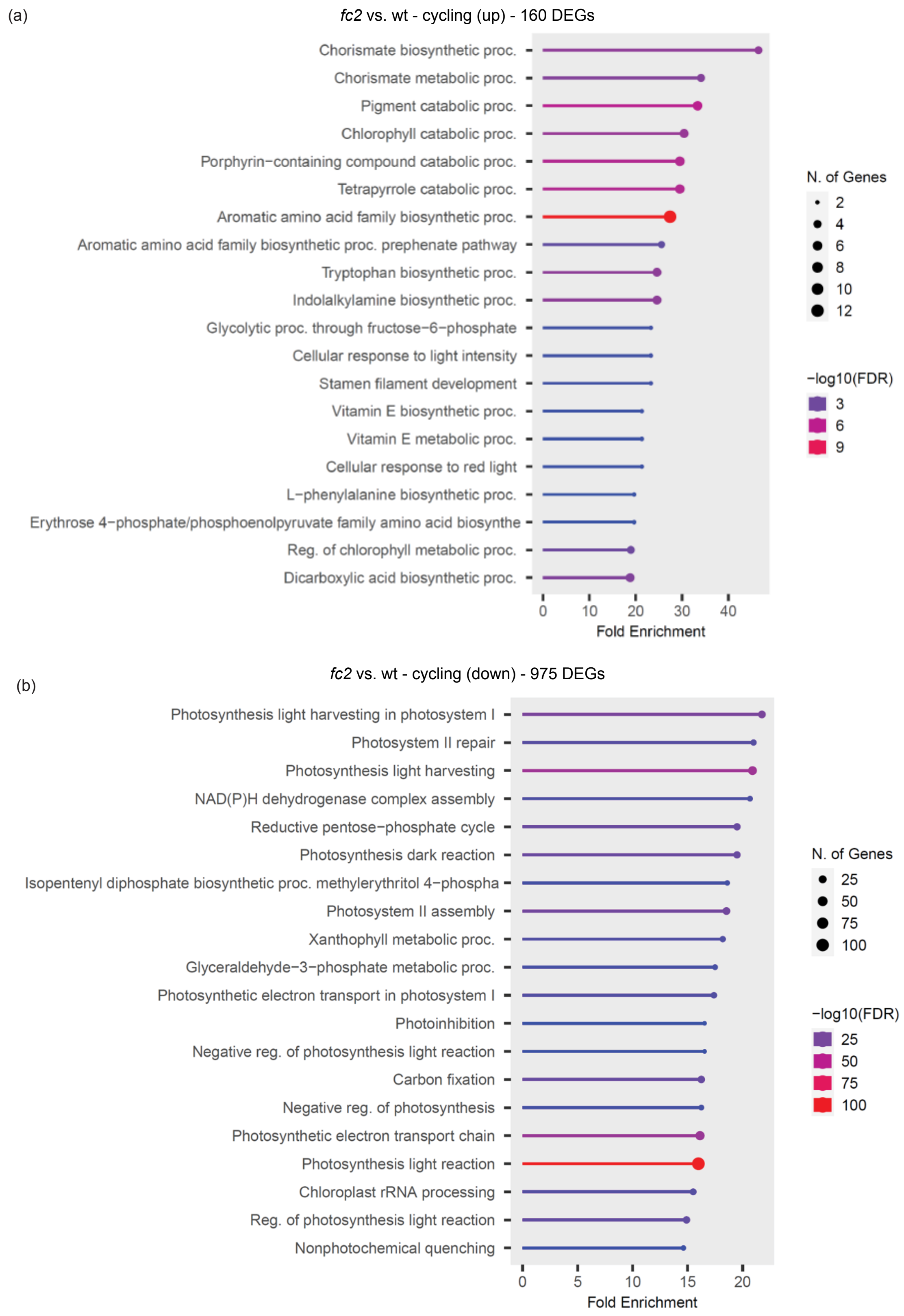

Figure S10. Gene ontology analyses of differentially expressed plastid protein-encoding genes in *fc2* mutants.

Shown are gene ontology (GO) term enrichment analyses of the identified differentially expressed plastid protein encoding genes (PPEGs) in this study. (a) GO-term enrichment from the 160 up-regulated PPEGs from *fc2* vs. wt under 16h light/8h dark diurnal cycling light conditions (cycling). (b) GO-term enrichment from the 975 down-regulated PPEGs from *fc2* vs. wt - cycling. GO-term enrichment analyses were performed using ShinyGO 0.80 with an FDR cutoff of $p \leq 0.01$. x-axes indicate fold-enrichment. The ball size indicates number of genes. The line colors represent FDR values.

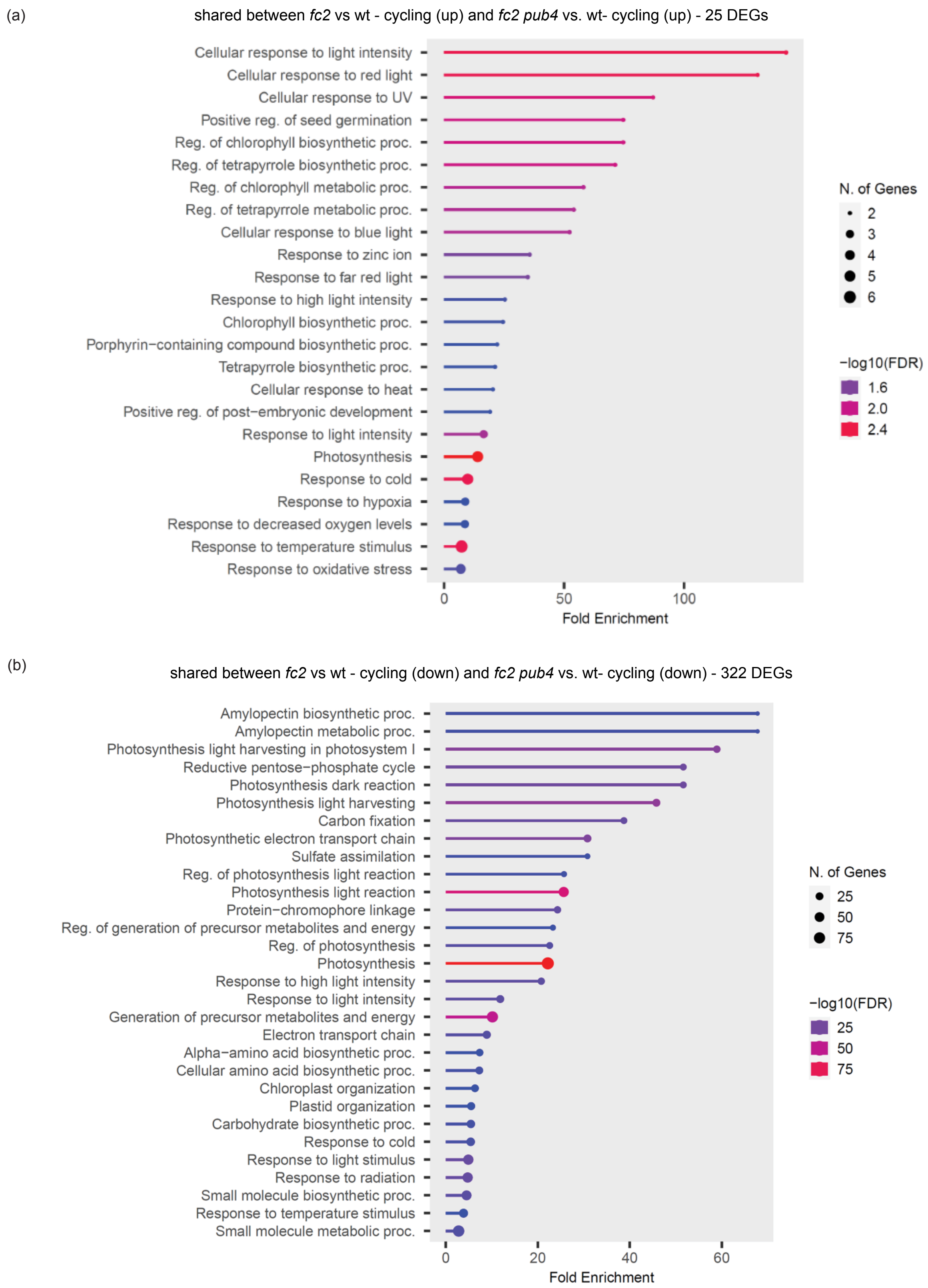

Figure S11. Gene ontology analyses of differentially expressed plastid protein-encoding genes shared between *fc2* and *fc2 pub4* mutants.

Shown are gene ontology (GO) term enrichment analyses of the identified differentially expressed plastid protein encoding genes (PPEGs) in this study. (a) GO-term enrichment from the shared 25 up-regulated PPEGs between *fc2* vs. wt – cycling and *fc2 pub4* – cycling. (b) GO-term enrichment from the shared 322 down-regulated PPEGs between *fc2* vs. wt – cycling and *fc2 pub4* – cycling. GO-term enrichment analyses were performed using ShinyGO 0.80 with an FDR cutoff of $p \leq 0.01$. x-axes indicate fold-enrichment. The ball size indicates number of genes. The line colors represent FDR values.

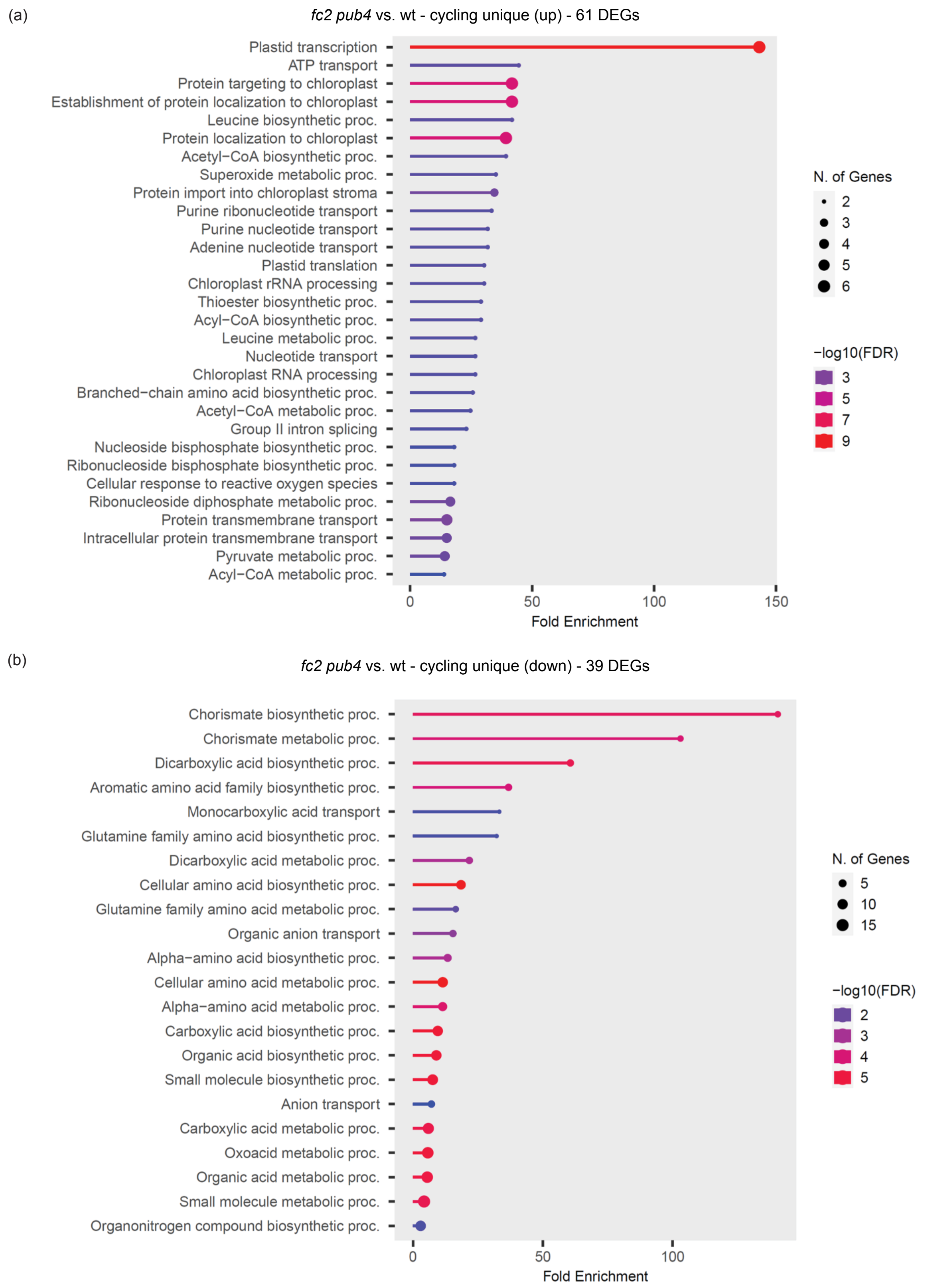

Figure S12. Gene ontology analyses of differentially expressed plastid protein-encoding genes unique to *fc2 pub4* mutants.

Shown are gene ontology (GO) term enrichment analyses of the identified differentially expressed plastid protein encoding genes (PPEGs) in this study. (a) GO-term enrichment from the unique 61 up-regulated PPEGs from *fc2 pub4* – cycling. (b) GO-term enrichment from the unique 39 down-regulated PPEGs from *fc2 pub4* – cycling. GO-term enrichment analyses were performed using ShinyGO 0.80 with an FDR cutoff of $p \leq 0.01$. x-axes indicate fold-enrichment. The ball size indicates number of genes. The line colors represent FDR values.

(a)

Plastid encoded chloroplast gene expression (Log2FC values)				
			fc2 vs. wt - cycling	*fc2 pub4* vs. wt - cycling
PSI	ATCG00350	psaA	-0.16	0.12
	ATCG00340	psaB	-0.42	-0.17
	ATCG01060	psaC	0.22	0.21
	ATCG00510	psaI	-0.52	-0.47
	ATCG00630	psaJ	-0.86	-0.16
PSII	ATCG00020	psbA	0.09	-0.32
	ATCG00680	psbB	0.19	-0.09
	ATCG00280	psbC	0.33	0.12
	ATCG00270	psbD	0.24	-0.10
	ATCG00580	psbE	-0.29	0.04
	ATCG00570	psbF	1.94	1.36
	ATCG00430	psbG	-0.13	0.02
	ATCG00710	psbH	0.37	0.36
	ATCG00080	psbI	-1.23	-0.96
	ATCG00550	psbJ	-0.10	-0.23
	ATCG00070	psbK	-1.03	-0.49
	ATCG00560	psbL	-0.25	0.52
	ATCG00220	psbM	0.08	0.76
	ATCG00700	psbN	0.37	0.24
	ATCG00690	psbT	-0.55	0.44
NDH complex	ATCG01100	ndhA	0.21	0.07
	ATCG00440	ndhC	-1.22	-1.17
	ATCG01050	ndhD	-0.74	-0.14
	ATCG01070	ndhE	-0.19	-0.20
	ATCG01010	ndhF	-0.73	-0.21
	ATCG01080	ndhG	-0.68	-0.30
	ATCG01110	ndhH	0.13	0.58
	ATCG01090	ndhI	0.07	0.29
	ATCG00420	ndhJ	-0.68	-0.39
ATP synthase	ATCG00120	atpA	-0.76	-0.33
	ATCG00480	atpB	-0.85	-0.59
	ATCG00470	atpE	-0.75	-0.38
	ATCG00130	atpF	-0.24	-0.13
	ATCG00140	atpH	0.27	-0.33
	ATCG00150	atpI	0.05	0.23
Cyt b6f complex	ATCG00540	petA	-0.72	-0.21
	ATCG00720	petB	-0.23	0.00
	ATCG00730	petD	-0.18	-0.19
	ATCG00600	petG	-1.49	0.09
RUBISCO	ATCG00490	rbcL	-0.35	-0.25

(b)

Nuclear encoded chloroplast gene expression (Log2FC values)				
			fc2 vs. wt - cycling	*fc2 pub4* vs. wt - cycling
PSI	AT4G02770	psaD1	-1.32	-0.29
	AT1G03130	psaD2	-1.55	-0.46
	AT4G28750	psaE1	-1.43	-0.37
	AT2G20260	psaE2	-1.19	-0.10
	AT1G31330	psaF	-1.42	-0.37
	AT1G55670	psaG	-1.43	-0.40
	AT3G16140	psaH1	-1.22	-0.23
	AT1G52230	psaH2	-1.59	-0.48
	AT1G30380	psaK	-1.61	-0.37
	AT4G12800	psaL	-1.43	-0.35
	AT5G64040	psaN	-1.49	-0.44
	AT1G08380	psaO	-1.59	-0.46
	AT2G46820	psaP	-1.42	-0.52
PSII	AT5G66570	psbO1	-1.11	-0.27
	AT3G50820	psbO2	-0.97	-0.23
	AT1G06680	psbP1	-1.29	-0.38
	AT2G30790	psbP2	-0.80	-0.61
	AT4G21280	psbQ1	-1.55	-0.53
	AT4G05180	psbQ2	-1.60	-0.42
	AT1G79040	psbR	-1.06	-0.12
	AT1G44575	psbS	-1.08	-0.24
	AT3G21055	psbTN	-1.40	-0.16
	AT2G30570	psbW	-1.16	-0.31
	AT2G06520	psbX	-1.19	-0.35
	AT1G67740	psbY	-1.13	-0.28
NDH complex	AT1G15980	ndh48	-1.14	-0.38
	AT1G64770	ndh45	-0.94	-0.13
	AT5G43750	ndh18	-1.38	-0.41
	AT3G16250	ndf4	-1.12	-0.36
	AT1G18730	ndf6	-1.28	-0.34
ATP synthase	AT4G04640	atpC1	-0.96	-0.26
	AT4G09650	atpD	-1.16	-0.46
Cyt b6f complex	AT4G03280	petC	-1.14	-0.20
	AT1G76100	petE1	-1.15	-0.26
	AT1G20340	petE2	-1.30	-0.31
	AT2G26500	petM	-0.93	-0.14
Light harvesting complex	AT3G54890	lhca1	-1.66	-0.38
	AT3G61470	lhca2	-1.47	-0.32
	AT1G61520	lhca3	-1.48	-0.42
	AT3G47470	lhca4	-1.71	-0.53
	AT1G19150	lhca6	-1.58	-0.46
	AT1G29920	lhcb1.2	-2.03	-0.28
	AT2G34430	lhcb1.4	-3.29	-0.78
	AT2G05070	lhcb2.2	-1.84	-0.33
	AT3G27690	lhcb2.3	-2.85	-0.71
	AT5G54270	lhcb3	-1.81	-0.58
	AT5G01530	lhcb4.1	-1.45	-0.37
	AT3G08940	lhcb4.2	-1.83	-0.58
	AT2G40100	lhcb4.3	-2.13	-0.10
	AT4G10340	lhcb5	-1.71	-0.53
	AT1G15820	lhcb6	-1.83	-0.59

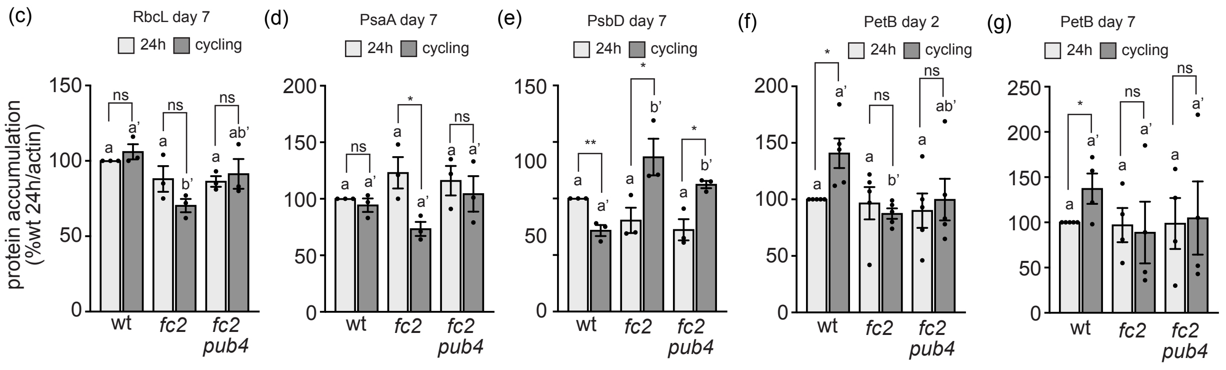

Figure S13. Relative expression of plastid protein-encoding genes during singlet oxygen stress.

Relative expression of select (a) plastid-encoded and (b) nuclear encoded (right column) chloroplast proteins in *fc2* and *fc2 pub4,* grown in 16h light/8h dark diurnal (cycling) light conditions, compared to wt. Shown are Log_2 Fold Change values from the DESeq2 analysis of the included RNA-seq data set. Values shaded in blue pass the significance cutoff (padj ≤ 0.01), and values in red font do not. The left columns indicate the protein complexes in which these gene products reside. Immunoblot analysis of selected plastid-encoded proteins (c) RbcL, (d) PsaA, (e) PsbD, and (f) and (g) PetB. from leaves of three-week-old plants grown in constant (24h) light conditions or grown in 24h light conditions and shifted to 16h light/8h dark diurnal (cycling) light conditions for two (F) or seven (C, D, E, and G) days. Shown are mean values compared to actin levels (+/- SEM) and normalized to wt in 24h light conditions (n ≥ 3 leaves from separate plants). Statistical analyses were performed using one-way ANOVA tests, and the different letters above the bars indicate significant differences within data sets determined by Tukey-Kramer post-tests (P ≤ 0.05). Separate analyses were performed for the different light conditions, and the significance for the cycling light condition is denoted by letters with a prime symbol (′). Statistical analyses of genotypes between conditions were performed by student's t-tests (*, $P \leq 0.05$; ns, P ≥ 0.05). Closed circles indicate individual data points.

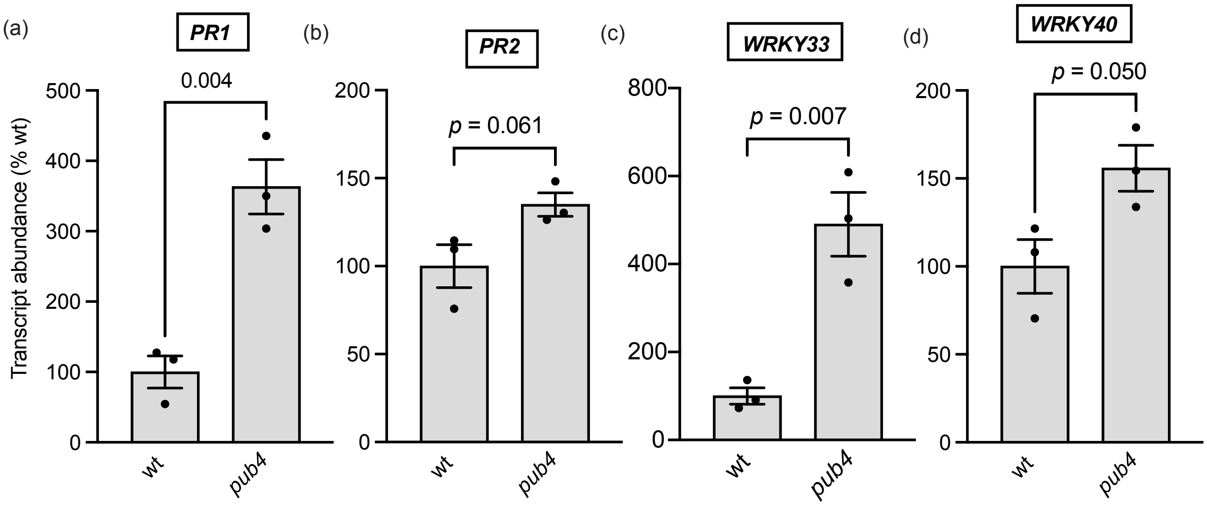

Figure S14. Analysis of salicylic acid response marker gene expression in the single *pub4* mutant. Shown are transcript profiles from 21-day-old plants as measured by RT-qPCR. (a-d)Transcript levels of salicylic acid (SA)-response genes (*PR1*, *PR2*, *WRKY33*, and *WRKY40*) in plants grown under 24h constant light conditions. Shown are mean values compared to *ACTIN2* expression (+/- SEM) and normalized to wt (n = 3 plants [total above ground tissue]). Statistical analyses were performed using a student's t-test and *p* values are indicated in each graph. Closed circles indicate individual data points.

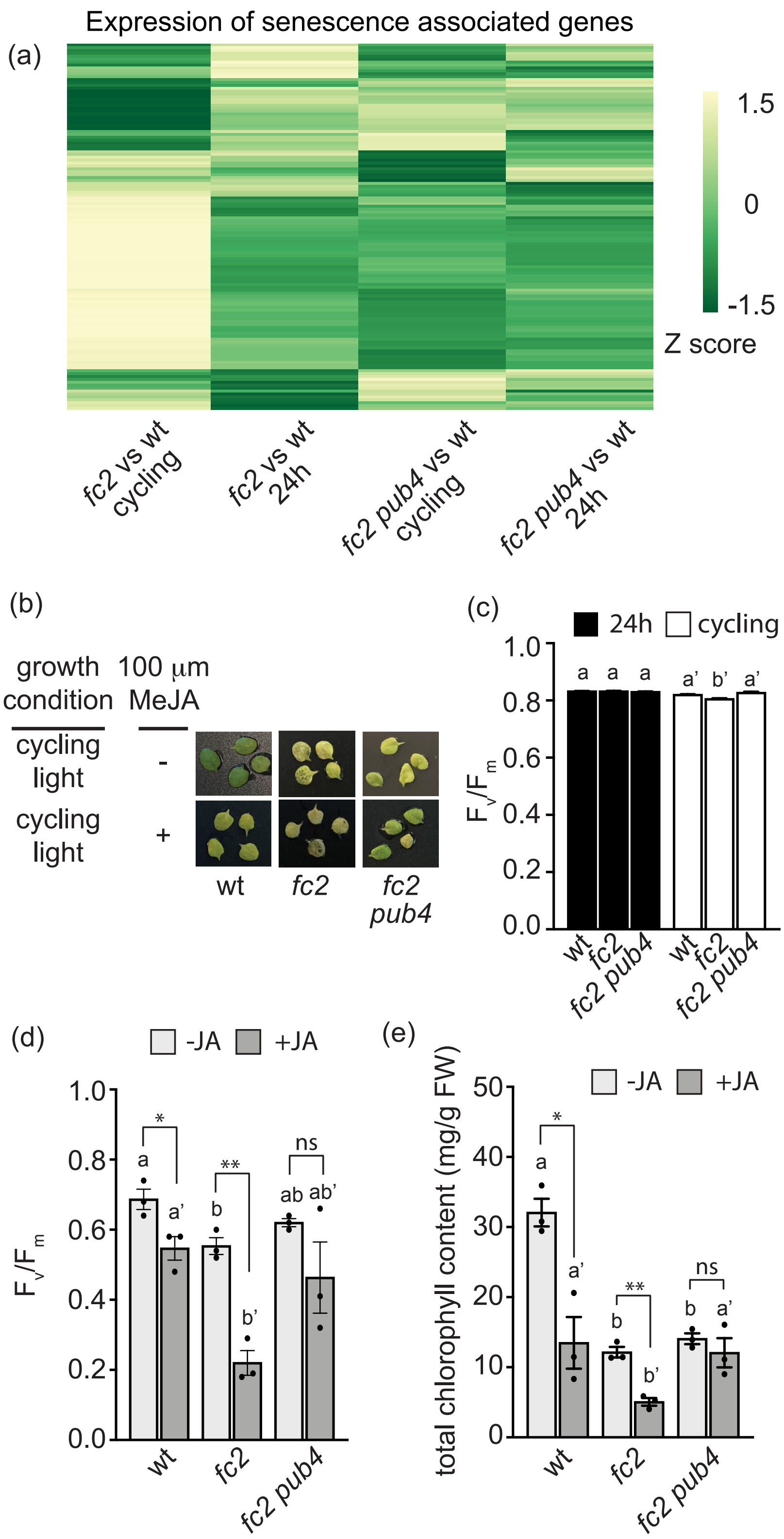

<u>Figure S15. Activation of senescence pathways is blocked by the *pub4* mutation.</u>

Heatmap showing the expression levels of 128 senescence-associated genes (GO-term "leaf senescence," GO:0010150) in *fc2* and *fc2 pub4* (relative to the wt), under constant (24h) light and 16h light/8h dark (cycling) light conditions. The gene expression data are Log_2 Fold Change values derived from DESeq2 analysis of the included RNA-seq data set. Genes that are up-regulated or down-regulated in comparison to wt are colored yellow or green, respectively. (b) Representative images of detached 3rd and 4th rosette leaves from plants grown in 24h light conditions plus two days of cycling light conditions. The leaves were incubated in the dark with water (control conditions) or with 100 μM methyl jasmonate (MeJA) for 3 days. Measurement of the maximum quantum efficiency of photosystem II (F_v/F_m) of (c) control leaves (left in light without JA) or (d) leaves from panel b. (e) Total chlorophyll content of leaves in panel b. Statistical analyses were performed using one-way ANOVA tests, and the different letters above the bars indicate significant differences within data sets determined by Tukey-Kramer post-tests ($P \leq 0.05$). Separate analyses were performed in c for each light condition and in d and e for the different treatments. The significance for cycling light samples in c and for the JA treatment in d and e are denoted by letters with a prime symbol (′). Statistical analyses of genotypes between conditions were performed by student's t-tests (*, $P \leq 0.05$; **, $P \leq 0.01$; ns, $P \geq 0.05$). n = 3 whole leaves from separate plants. Error bars = +/- SEM. Closed circles indicate individual data points.
