# Supplementary material for: Transcript profiling of plastid ferrochelatase two mutants reveals that chloroplast singlet oxygen signals lead to global changes in RNA profiles and are mediated by Plant U-Box 4
Source: BMC Plant Biol. 2025 Jun 3;25:747. doi: 10.1186/s12870-025-06703-7 (PMC12131553; doi:10.1186/s12870-025-06703-7)
Supplement: Supplementary file 3 — Additional file 3: Supplemental information, Table S8. Table S8. Comparisons between whole transcriptome datasets. [file 12870_2025_6703_MOESM3_ESM.pdf]

Supplemental information, Table S8.

This file contains the following Supplemental Table on a separate page:

Table S8. Comparisons between whole transcriptome datasets.

Table S8. Comparisons between whole transcriptome datasets.

	**This study.**<br>***fc2* in cycling light**	**Etiolated *fc2* seedlings [1]**	**Adult *flu* mutants [2]**	**Adult *ch1* mutant [3]**	***flu* seedlings (early singlet oxygen response genes (ESORGS) [4]**
**Toal DEGs**	1549 (up)<br>822 (down)	185 (up)<br>241 (down)	588 (up)<br>77 (down)	520 (up)<br>370 (down)	125 (up)
**Overlap with current data set**		86 (up)<br>89 (down)	275 (up)<br>41 (down)	281 (up)<br>126 (down)	67 (up)
**% overlap**		46% (up)<br>37% (down)	47% (up)<br>53% (down)	54% (up)<br>34% (down)	54% (up)
**Hypergeometric test p-value**		4.38e-46 (up)<br>8.52e-61 (down)	2.98e-150 (up)<br>1.89e-36 (down)	8.31e-176 (up)<br>1.99e-81 (down)	3.30e-41 (up)
**Overlap with PUB4-dependent DEGs identified in this study**	1369 (up)<br>769 (down)	70 (up)<br>86 (down)	249 (up)<br>39 (down)	240 (up)<br>119 (down)	61 (up)
**% overlap**	88% (up)<br>94% (down)	38% (up)<br>36% (down)	42% (up)<br>51% (down)	46% (up)<br>32% (down)	49% (up)
**Hypergeometric test p-value**		3.85e-34 (up)<br>9.72e-60 (down)	4.09e-136 (up)<br>1.01e-34 (down)	1.42e-141 (up)<br>5.61e-77 (down)	1.30E-37 (up)

References

1. Woodson JD, Joens MS, Sinson AB, Gilkerson J, Salome PA, Weigel D, Fitzpatrick JA, Chory J: **Ubiquitin facilitates a quality-control pathway that removes damaged chloroplasts**. *Science* 2015, **350**(6259):450-454.
2. op den Camp RG, Przybyla D, Ochsenbein C, Laloi C, Kim C, Danon A, Wagner D, Hideg E, Gobel C, Feussner I *et al*: **Rapid induction of distinct stress responses after the release of singlet oxygen in Arabidopsis**. *Plant Cell* 2003, **15**(10):2320-2332.
3. Ramel F, Ksas B, Akkari E, Mialoundama AS, Monnet F, Krieger-Liszkay A, Ravanat JL, Mueller MJ, Bouvier F, Havaux M: **Light-induced acclimation of the Arabidopsis *chlorina1* mutant to singlet oxygen**. *Plant Cell* 2013, **25**(4):1445-1462.
4. Dogra V, Duan J, Lee KP, Lv S, Liu R, Kim C: **FtsH2-Dependent Proteolysis of EXECUTER1 Is Essential in Mediating Singlet Oxygen-Triggered Retrograde Signaling in *Arabidopsis thaliana***. *Front Plant Sci* 2017, **8**:1145.
