# Supplementary material for: Transcript profiling of plastid ferrochelatase two mutants reveals that chloroplast singlet oxygen signals lead to global changes in RNA profiles and are mediated by Plant U-Box 4
Source: BMC Plant Biol. 2025 Jun 3;25:747. doi: 10.1186/s12870-025-06703-7 (PMC12131553; doi:10.1186/s12870-025-06703-7)
Supplement: Supplementary file 5 — Additional file 5: Supplemental information, Tables S27 and S28. Table S27. Mutant plant lines used in study, Table S28. List of primers used for RT-qPCR and Genotyping. [file 12870_2025_6703_MOESM5_ESM.pdf]

Supplemental information, Tables S27 and S28

This file contains the following Supplemental Tables, each on a separate page:

Table S27. Mutant lines used in this study

Table S28. Primers used in study

Table S27. Mutant lines used in this study

Mutant	Gene	mutation	notes	ref
fc2-1	*PLASTID FERROCHELATASE 2, FC2, AT2G30390*	GABI_766H08<br>T-DNA in 5'UTR	Sulfadiazin^r	[1]
pub4-6	*PLANT U-BOX 4, PUB4, AT2G23140*	Point mutation (c9847535t) leading to amino acid substitution (G255R)		[2]

Table S28. Primers used in study

**RT-qPCR primers**		
AT3G18780 (ACTIN2)	For. JP199	GCACTTGCACCAAGCAGCAT
	Rev. JP200	CCTTTCAGGTGGTGCAACGAC
AT3G56710 (SIB1)	For. JP589	CAACCGGAGCCCATCTATT
	Rev. JP590	GGAGAAAGGTTGTGGTCGTC
AT3G44880 (PAO)	For. WLO1690	TTGGATCCGAATGTGCCAAC
	Rev. WLO1691	TCAAAGGCTGCCCATTTCTG
AT5G13630 (CHLH)	For. WLO1692	TGCAAGCTTTGGAAGGCAAG
	Rev. WLO1693	ACTTGCCATTGCTGCTGTTG
AT1G72520 (LOX4)	For. WLO1696	TTGCGTCTCCGTTTCATTCG
	Rev. WLO1697	ACAACGCCGCTTGAGTTAAC
AT3G25780 (AOC3)	For. WLO1698	ACTCGGCAAGAAACCAACAG
	Rev. WLO1699	TGATTCCCACGCGTTTCTTG
AT1G74710 (EDS16)	For. WLO1704	TGGTGCACCAGCTTTTATCG
	Rev. WLO1705	AAAGCTTCACTGCAGACACC
AT5G37780 (CAM1)	For. WLO1712	ATGGAAACGGCACTATCGAC
	Rev. WLO1713	TTGTCGAAAACCCTGAAGGC
AT2G26300 (GPA1)	For. WLO1714	TGCAAGAGTTCGCACAACTG
	Rev. WLO1715	TCCACCCACGTCAAACAATC
AT1G56650 (*PAP1*/*MYB75*)	For. WLO1959	GCGAAAAGGTGCTTGGACTAC
	For. WLO1960	ACTTGGTGCCATTTGCCTTC
AT2G14610 (*PR1*)	For. WLO1771	GTGCTCTTGTTCTTCCCTCGA
	Rev. WLO1772	CCCACGAGGATCATAGTTGCA
AT2G38470 (*WRKY33*)	For. WLO1632	CCAAACCGAGACTCGTCCAA
	Rev. WLO1633	TGCACTACGATTCTCGGCTC
AT1G80840 (*WRKY40*)	For. WLO1812	ACAACCATCCAATGCCATCG
	Rev. WLO1813	TCTTCTGTTTGCTGCAACGG
AT3G52430 (*PAD4*)	For. WLO1694	ACGAGTGAGTTGCAAGCTTC
	Rev. WLO1695	GCAATTTGCGGTGTTGCATG
At2G43620 (*Chitinase*)	For. WLO2872	TGAGCAAAGTCGGAAATGGC
	Rev. WLO2873	AACATAGCGGCAATCTCACG
AT1G77380 (*AAP3*)	For. WLO2874	CCGCTTGAATGTCTTCAGGTTG
	Rev. WLO2875	ACACCGTCAATGGCCAAAAC
AT5G46050 (*PTR3*)	For. WLO2876	TGTTGGGCACTCCGTTTTAC
	Rev. WLO2877	TGCGAAATGAAGCCACGATG
At3G61190 (*BAP1*)	For. JP338	ATTGATGGATACGGTGGCCG
	Rev. JP339	CAGACCCCAAACCGGAACTC
At5G64870 (*NOD*)	For. JP340	GCTGATGCTGCCTTCTATTCAA
	Rev. JP341	TGCGACAAGTCCCTCTGCA
At5G59820 (*ZAT12*)	For. JP344	GCGTTGGTTACACGCGCTT
	Rev. JP345	CTTCAACGTAGTCACCGTGGG
AT3G01500 (*CA1*)	For. JP209	TGTGTCCATCACACGTTCTGG
	Rev. JP210	GGACCACGAAGGCATCTCCT
At1G29910 (*LHCB1.2*)	For. JP197	GGACTTGCTTTACCCCGGTG
	Rev. JP198	TCGGTAGCAAGACCCAATGG
AT3G57260 (*PR2*)	For. WLO2226	GATCGTTGGAAATCGTGGTG
	Rev. WLO2227	TAGCTTTCCCTGGCCTTCTC

References

1. Woodson JD, Perez-Ruiz JM, Chory J: **Heme synthesis by plastid ferrochelatase I regulates nuclear gene expression in plants**. *Curr Biol* 2011, **21**(10):897-903.
2. Woodson JD, Joens MS, Sinson AB, Gilkerson J, Salome PA, Weigel D, Fitzpatrick JA, Chory J: **Ubiquitin facilitates a quality-control pathway that removes damaged chloroplasts**. *Science* 2015, **350**(6259):450-454.
