# Supplementary material for: Transcript profiling of plastid ferrochelatase two mutants reveals that chloroplast singlet oxygen signals lead to global changes in RNA profiles and are mediated by Plant U-Box 4
Source: BMC Plant Biol. 2025 Jun 3;25:747. doi: 10.1186/s12870-025-06703-7 (PMC12131553; doi:10.1186/s12870-025-06703-7)
Supplement: Supplementary file 6 — Additional file 6: Supplemental information, Methods S1 through S3. Methods 1. Comparison with additional singlet oxygen response datasets, Methods 2. Transcription factor analysis, Methods 3. RNA extraction, cDNA synthesis, and real-time quantitative PCR. [file 12870_2025_6703_MOESM6_ESM.pdf]

Supplemental information, Methods S1 through S3

This file contains the following Supplemental methods:

Methods S1. Comparison with additional singlet oxygen response datasets

Methods S2. Transcription factor analysis

Methods S3. RNA extraction, cDNA synthesis, and real-time quantitative PCR

Methods S1. Comparison with additional singlet oxygen response datasets

Differentially expressed genes (DEGs) identified in the *fc2* mutant during singlet oxygen ($^1O_2$)-producing cycling light conditions ("*fc2* vs. wt – cycling") in this study were compared to $^1O_2$-related DEGs identified in previous studies using etiolated *fc2* seedlings [1] (ATH1 microarray), *flu* seedlings [2] (RNA-seq), adult *flu* plants [3] (ATH1 microarray), and adult *ch1* plants [4] (CATv5 Microarray). Here, we used DEGs identified in these datasets from a previous meta-analysis [5] that applied cutoffs (fold change +/- 2, *padj* ≤ 0.05) and only considered genes that could be analyzed in all methods used (Affymetrix GeneChip Arabidopsis ATH1 and CATv5 Microarrays and RNA-seq) (19,895 genes). We applied the same cutoffs and gene consideration to the current dataset, comparing *fc2* with wt under cycling light conditions (1549 up-regulated and 822 down-regulated DEGs). To identify DEGs in these additional datasets that are potentially regulated by PUB4, we first identified DEGs in "*fc2 pub4* vs. wt – cycling" with the same cutoffs. DEGs unique to "*fc2* vs. wt – cycling" (not shared with "*fc2 pub4* vs. wt – cycling") were considered to be reversed by the *pub4* mutation and were compared to the DEGs in the additional datasets. Etiolated *fc2* seedlings, etiolated seedlings exposed to light for two hours (wt vs. *fc2*) [1]; adult *flu* mutants (plants incubated in dark for 8 hours, and re-exposed to light for one hour (wt vs. *flu*) [3]; adult *ch1* mutants, *ch1* plants shifted from 180 (low light) to 1,000 (excess light) µmol photons m^{-2} sec^{-1} for 2 days (low light vs. excess light) [4]; *flu* seedlings (early singlet oxygen response genes (ESORGS)), 5-day-old *flu* seedlings incubated in the dark for 4 hours and re-exposed to light for 30 and 60 min (light treatment vs. dark only) [2].

Methods S2. Transcription factor analysis

Data used in TF analysis.

To perform transcription factor (TF) enrichment analyses, DESeq2 was used to identify differentially expressed genes (DEGs) (from all four pairwise comparisons: *fc2* vs. wt – 24h, *fc2* vs. wt – cycling, *fc2 pub4* vs. wt – 24h, *fc2 pub4* vs. wt – cycling) with the following cutoff criteria: $\log_2$FC +/- ≥ 1, adjusted p-value ≤ 0.01. DEGs that passed these cutoff criteria (3121 genes) were compared to a list of known transcription factors in Arabidopsis (representing 1717 unique loci) from PlantTFDB to identify 254 TFs (http://planttfdb.gao-lab.org/index.php?sp=Ath) [6-8].

Heatmap generation.

The expression of these 254 genes were compared across all four pairwise data sets to produce an expression matrix (**Table S29**, which was used to generate a heatmap using the heat mapper package (https://github.com/WishartLab/heatmapper) on http://www.heatmapper.ca/ [9].

TF enrichment.

Differentially expressed TFs (DETFs) were identified from DEG lists using PlantTFDB, were compiled (**Table S13 and S14**) and the counts for each family of TF were determined (**Table S15**). A hypergeometric test followed by the Benjamini–Hochberg method was used to determine the

False Discovery Rate (FDR) of each TF family identified (**Table S16**). A cutoff (FDR < 0.05) was applied and the -log10(FDR) was calculated for each TF family that passed this cutoff.

TF Gene Ontology Analysis.

Gene ontology analysis was performed using Database for Annotation, Visualization and Integrated Discovery (DAVID) 2021 (https://david.ncifcrf.gov/) [10, 11]. Lists of DETFs from cycling light-exposed plants (*fc2* vs. wt - cycling and *fc2 pub4* vs. wt - cycling) were used to identify the enrichment of Biological GO terms represented both in full DETF lists and sub lists of the different TF families that were significantly enriched in our TF enrichment analysis (**Table S17**). The reported GO terms represent the top 10 statistically significant (FDR ≤ 0.05) GO terms excluding terms relating to transcriptional regulation.

Transcription factor enrichment analysis (TFEA).

A list of TFs whose targets were overrepresented in DEG lists was obtained from the TF enrichment tool on http://plantregmap.gao-lab.org/tf_enrichment.php (**Table S18**) and the counts for each TF family represented were determined (**Table S15**). A hypergeometric test followed by the Benjamini–Hochberg method was used to determine the False Discovery Rate (FDR) of each TF family identified (**Table S19**). A cutoff (FDR ≤ 0.05) was applied and the -log10(FDR) was calculated for each TF family that passed this cutoff.

Methods S3. RNA extraction, cDNA synthesis, and real-time quantitative PCR

Measurement of steady-state RNA transcripts were made as previously described [12]. The RNeasy Plant Mini Kit (Qiagen) was used to extract total RNA from whole seedlings. Next, cDNA was synthesized using the Maxima first strand cDNA synthesis kit for RT-qPCR with DNase (Thermo Scientific) following the manufacturer's instructions. Real-time PCR was performed using the SYBR Green Master Mix (BioRad) with the SYBR Green fluorophore and a CFX Connect Real Time PCR Detection System (BioRad). The following 2-step thermal profile was used in all RT-qPCR: 95 °C for 3 min, 40 cycles of 95 °C for 10s and 60 °C for 30s. *ACTIN2* expression was used as a standard to normalize all gene expression data. **Table S28** lists the primers used.

References

1. Woodson JD, Joens MS, Sinson AB, Gilkerson J, Salome PA, Weigel D, Fitzpatrick JA, Chory J: **Ubiquitin facilitates a quality-control pathway that removes damaged chloroplasts**. *Science* 2015, **350**(6259):450-454.
2. Dogra V, Duan J, Lee KP, Lv S, Liu R, Kim C: **FtsH2-Dependent Proteolysis of EXECUTER1 Is Essential in Mediating Singlet Oxygen-Triggered Retrograde Signaling in *Arabidopsis thaliana***. *Front Plant Sci* 2017, **8**:1145.
3. op den Camp RG, Przybyla D, Ochsenbein C, Laloi C, Kim C, Danon A, Wagner D, Hideg E, Gobel C, Feussner I *et al*: **Rapid induction of distinct stress responses after the release of singlet oxygen in Arabidopsis**. *Plant Cell* 2003, **15**(10):2320-2332.
4. Ramel F, Ksas B, Akkari E, Mialoundama AS, Monnet F, Krieger-Liszkay A, Ravanat JL, Mueller MJ, Bouvier F, Havaux M: **Light-induced acclimation of the Arabidopsis *chlorina1* mutant to singlet oxygen**. *Plant Cell* 2013, **25**(4):1445-1462.
5. Tano DW, Kozlowska MA, Easter RA, Woodson JD: **Multiple pathways mediate chloroplast singlet oxygen stress signaling**. *Plant Molecular Biology* 2023, **111**:167-187.
6. Jin J, Zhang H, Kong L, Gao G, Luo J: **PlantTFDB 3.0: a portal for the functional and evolutionary study of plant transcription factors**. *Nucleic Acids Res* 2014, **42**(Database issue):D1182-1187.
7. Jin J, He K, Tang X, Li Z, Lv L, Zhao Y, Luo J, Gao G: **An Arabidopsis Transcriptional Regulatory Map Reveals Distinct Functional and Evolutionary Features of Novel Transcription Factors**. *Mol Biol Evol* 2015, **32**(7):1767-1773.
8. Jin J, Tian F, Yang DC, Meng YQ, Kong L, Luo J, Gao G: **PlantTFDB 4.0: toward a central hub for transcription factors and regulatory interactions in plants**. *Nucleic Acids Res* 2017, **45**(D1):D1040-D1045.
9. Babicki S, Arndt D, Marcu A, Liang Y, Grant JR, Maciejewski A, Wishart DS: **Heatmapper: web-enabled heat mapping for all**. *Nucleic acids research* 2016, **44**:W147-W153.
10. Huang da W, Sherman BT, Lempicki RA: **Systematic and integrative analysis of large gene lists using DAVID bioinformatics resources**. *Nat Protoc* 2009, **4**(1):44-57.
11. Sherman BT, Hao M, Qiu J, Jiao X, Baseler MW, Lane HC, Imamichi T, Chang W: **DAVID: a web server for functional enrichment analysis and functional annotation of gene lists (2021 update)**. *Nucleic Acids Res* 2022, **50**(W1):W216-W221.
12. Alamdari K, Fisher KE, Sinson AB, Chory J, Woodson JD: **Roles for the chloroplast-localized PPR Protein 30 and the "Mitochondrial" Transcription Termination Factor 9 in chloroplast quality control**. *Plant J* 2020, **103**(3):735-751.
